# Epidemic forecast and preparedness for explosive-cerebrospinal meningitis outbreak in Nigeria using the preventive vaccination strategy

**DOI:** 10.4102/ajlm.v12i1.2086

**Published:** 2023-11-17

**Authors:** Iseimokumo C. Peletiri, Rosemary C. Nwachukwu, Diweni C. Peletiri, Esther Q. Onoja, Charity T. Tulagha, Ikaprite I. Igbalibo, Grace M. Ayanbimpe, Eugene I. Ikeh

**Affiliations:** 1 Department of Medical Microbiology, Faculty of Clinical Sciences, College of Health Sciences, University of Jos, Jos, Plateau State, Nigeria; 2 Medical Microbiology & Parasitology Laboratories, National Hospital, Abuja, Federal Capital Territory, Nigeria; 3 Medical Microbiology Laboratory, Department of Medical Laboratory Services, Federal Medical Centre, Yenagoa, Bayelsa State, Nigeria; 4 Medical Biotechnology Department, National Biotechnology Development Agency, Abuja, Federal Capital Territory, Nigeria; 5 Hematology and Blood Transfusion Laboratory Department, National Hospital, Abuja, Federal Capital Territory, Nigeria; 6 Nursing Services Department, Diete-Koki Memorial Hospital, Opolo, Yenagoa, Bayelsa State, Nigeria

**Keywords:** meningitis epidemic forecast, explosive-cerebrospinal meningitis outbreak, reactive vaccination strategy, preventive vaccination strategy, African meningitis belt, Nigeria

## Abstract

**Background:**

Within the African meningitis belt, yearly outbreaks of cerebrospinal meningitis (CSM), with incidence rates of 10–100 cases per 100 000 population, are typically punctuated by explosive epidemics occurring every 8–12 years, with incidence rates that can exceed 1000 cases per 100 000 population. From 1928 to 2018, Nigeria recorded the highest number (21%) of cases in the region. The reactive vaccination strategy, a protocol with major drawbacks, has been the vaccination method utilised in Nigeria.

**Aim:**

This review highlights the need for governments within the African meningitis belt to start preparations against the next explosive CSM epidemic expected to occur between 2024 and 2028 using the preventive vaccination strategy.

**Methods:**

We performed a literature search on the Google Scholar search engine using relevant search strings and included studies and reports between 1905 and 2022 that met set criteria.

**Results:**

*Neisseria meningitidis* serogroups A, B, C, W135, X, and Y; *Haemophilus influenzae* serotypes a, b, c, e, and f; and *Streptococcus pneumoniae* serotypes 1, 4, 5, 6, 9, 19, 19F, and 20 were implicated as aetiologies. However, the reactive vaccination strategy was only used against *N. meningitidis* A or C, *H. influenzae* b, and pneumococcal conjugate vaccine. Between 2011 and 2017, a polysaccharide vaccine (ACW or ACYW) active against serogroups A, C, W and Y was used within the African meningitis belt for the first time. Varying genotypes of *N. meningitidis, H. influenzae and S. pneumoniae* were identified.

**Conclusion:**

Our results revealed a very high success rate for the preventive vaccination strategy.

**What this study adds:**

In order to ensure reductions in the morbidity and mortality associated with invasive CSM, the Federal Ministry of Health, Nigeria, should leverage existing knowledge of the circulating serogroups, serotypes, and genotypes of the primary bacterial aetiologies and commence the implementation of the preventive vaccination strategy.

## Introduction

**FIGURE 1:**
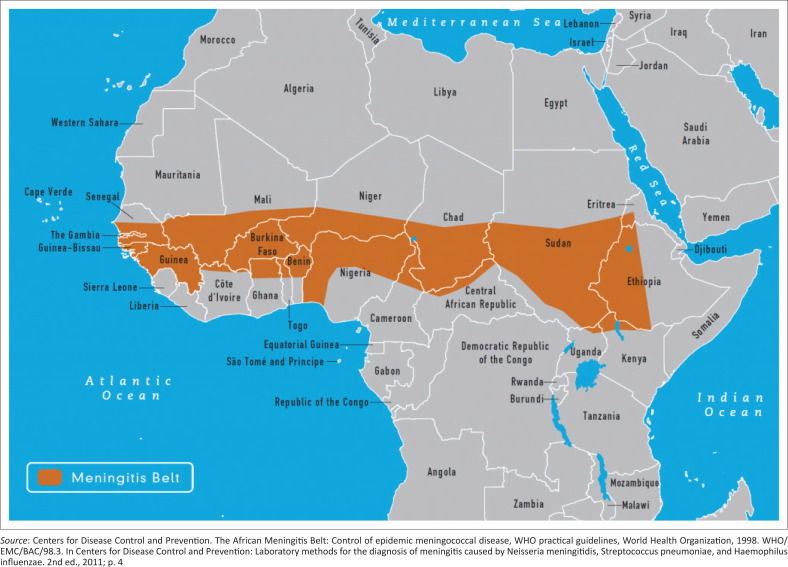
The African meningitis belt, 1998.

**FIGURE 2:**
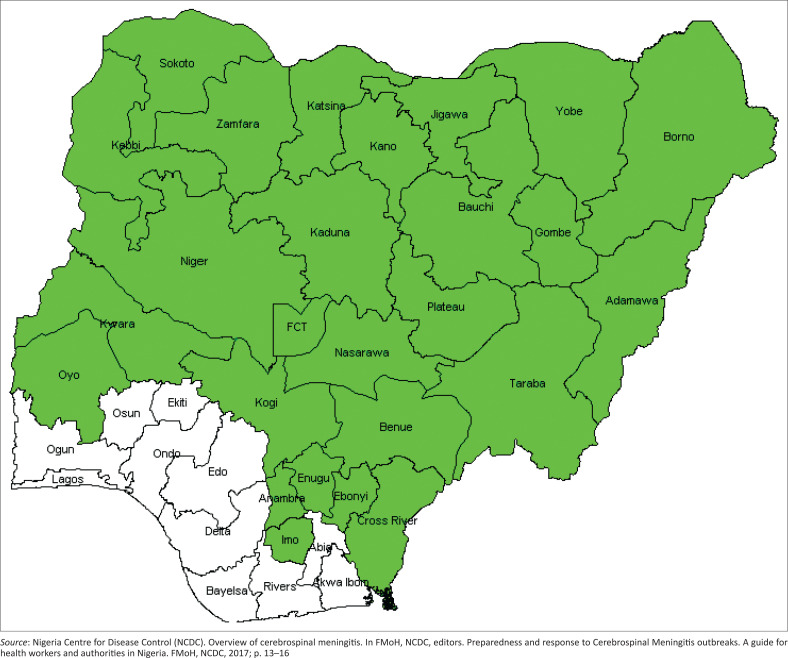
States within the meningitis belt in Nigeria, 2017.

The occurrence of large epidemics of meningococcal meningitis in Africa for a century led to the delineation of the African meningitis belt,^1^ comprising 26 countries: Benin, Burkina Faso, Burundi, Cameroon, Central African Republic, Chad, Côte d’Ivoire, Democratic Republic of the Congo, Eritrea, Ethiopia, The Gambia, Ghana, Guinea, Guinea-Bissau, Kenya, Mali, Mauritania, Niger Republic, Nigeria, Rwanda, Senegal, South Sudan, Sudan, Tanzania, Togo and Uganda (Figure 1).^2^ Since 1905, when the first meningitis outbreak was reported to have occurred in Zungeru, northern Nigeria, meningitis outbreaks have become an annual occurrence and a household menace in Nigeria.^3^ Of the 36 states and the Federal Capital Territory in Nigeria, 25 states, including the Federal Capital Territory, are now within the meningitis belt (Figure 2).^4^

Every year, a meningitis outbreak occurs in the dry season within the African meningitis belt. The outbreak typically occurs between December and June,^5^ with incidence rates of 10–100 cases per 100 000 population. These annual outbreaks are usually punctuated by explosive epidemics occurring every 8–12 years, with incidence rates that can exceed 1000 cases per 100 000 population.^6,7^ The 1996 cerebrospinal meningitis (CSM) outbreak was the most serious epidemic ever recorded in Nigeria, with 109 580 cases and 11 717 deaths, giving a case fatality rate (CFR) of 10.7%.^8^ From 1928 to 2018, Nigeria recorded the highest number of CSM cases (552 821 cases; 21% of the total) within the African meningitis belt.^9^

Historically, a few bacterial aetiologies have been implicated in the major epidemics recorded in Nigeria. Before the introduction of the meningococcal A conjugate vaccine, Nigeria encountered three major epidemics, all caused by *Neisseria meningitidis* serogroup A. These epidemics occurred in 1977 (1257 cases; CFR = 8.3%),^10^ 1996 (109 580 cases; CFR = 10.7%),^8^ and 2009 (55 626 cases; CFR = 4.1%).^11^ However, after the introduction of the meningococcal A conjugate vaccine, the aetiologic serogroup changed. Two epidemics in 2015 (6394 cases; CFR = 5.0%)^12^ and 2017 (5595 cases; CFR = 10.9%)^13^ were caused by *N. meningitidis* serogroup C. Vaccination efforts against these yearly meningitis outbreaks have been based on the ‘reactive vaccination strategy’, a methodology with identified major drawbacks such as delays in confirming outbreaks and deploying vaccines, difficulty in assessing some remote communities, limited capacity of health workers in the collection of cerebrospinal fluid (CSF) specimens, certainty of deaths during epidemics, as well as confirmed cases of survivors of CSM with sequelae, among others. With the looming spectre of a global explosive CSM epidemic, improving infectious disease (e.g., meningitis) forecasting continues to be a major priority of global health preparedness.^14,15,16^

Serotyping is vital in the development of vaccination strategies,^17^ as well as for the identification, containment and subsequent prevention of outbreaks. It also guides the determination of appropriate public health responses such as the administration of chemoprophylaxis for contacts of *Haemophilus influenzae* type b (Hib) cases or the administration of meningococcal conjugate versus serogroup B meningococcal (MenB) vaccine during outbreaks of *N. meningitidis.*^18,19,20^ Knowledge of the circulating bacterial genotypes are meant to establish if a disease outbreak is short term (local epidemiology) or long term (global epidemiology).^21^

This review aimed to highlight the major drawbacks of the reactive vaccination strategy, the benefits of the preventive vaccination strategy and the importance of serotyping as Nigerian as well as other governments and health authorities within the African meningitis belt prepare for the next explosive CSM epidemic expected to occur between 2024 and 2028.

## Methods

We performed a literature search on the Google Scholar search engine (https://scholar.google.com/) using the following search terms: ‘laboratory examination of cerebrospinal fluid (CSF)’; ‘laboratory diagnosis of meningitis’; ‘control of meningitis outbreak in the African meningitis belt’; ‘epidemic meningitis in sub-Saharan Africa’; ‘clinical features in adults with bacterial meningitis’; ‘*N. meningitidis* serogroups found in Nigeria’; ‘meningitis caused by *H. influenzae* serotypes in Nigeria’; ‘meningitis caused by *Streptococcus pneumoniae* serotypes in Nigeria’; ‘*N. meningitidis* conjugate vaccines’; ‘*H. influenzae* type b routine childhood vaccination’; ‘*S. pneumoniae* vaccination plan’; ‘immunisation programmes against *N. meningitidis*’; ‘immunisation programmes against meningococcal meningitis’; ‘immunisation programmes against meningitis caused by *H. influenzae*’; ‘immunisation programmes against pneumococcal meningitis’; ‘vaccination against meningitis outbreak’; ‘impact of vaccination during meningitis outbreak’; ‘*N. meningitidis* genotypes in Nigeria’; ‘*H. influenzae* genotypes in Nigeria’; *S. pneumoniae* genotypes in Nigeria’. The search was done from 2015–2022 with three active reviewers and four passive reviewers. The epidemic forecast was performed prospectively.

### Inclusion criteria

We included articles that reported results of laboratory examination of CSF and laboratory diagnosis of CSM, as well as articles that used a definite vaccination protocol either during or after a meningitis outbreak between 1905 and 2022. All articles that discussed the laboratory diagnosis of meningitis with results of the serogroups or serotypes of bacterial aetiologies of CSM within Nigeria were included. We also included articles that reported the circulating genotypes of the three major bacterial aetiologies (*N. meningitidis, H. influenzae*, and *S. pneumoniae*) in Nigeria. Finally, we included all the articles that reported the use of any of the vaccination strategies (reactive or preventive) globally.

### Exclusion criteria

We excluded articles that did not report on the serogroups, serotypes, or circulating genotypes of the bacterial aetiologies of meningitis within the meningitis belt of Nigeria. We also excluded articles that did not report on vaccination strategies.

### Forecast methodology

The epidemic forecast was performed prospectively based on credible information that explosive meningitis epidemics occur every 8–12 years with incidence rates that can exceed 1000 cases per 100 000 population.^6,7^ Since the last explosive epidemic was in 2017, by simple extrapolation, the next epidemic is expected to occur between 2024 and 2028.

### Forecast accuracy evaluation

The forecast accuracy evaluation method used relied on the documented evidence from Moore in 1992^6^ and the Centre for Disease Control and Prevention^7^ that explosive CSM epidemics occur every 8–12 years,^6,7^ with the last occurrence being in 2017 as recorded by Nigeria Centre for Disease Control.^13^ The Centre for Disease Control and Nigeria Centre for Disease Control are authorities responsible for the management of CSM globally and locally, hence the method we used for this forecast is justified.

## Results

Results generated from the Google Scholar search engine returned 68 articles that met the set criteria and were included in the final analysis. However, the results were grouped into four categories. The first category comprised studies that reported the laboratory diagnostic protocols used in the identification of bacterial aetiologies of CSM in the African meningitis belt. In low-resource countries like Nigeria, the laboratory diagnostic methods used are presumptive identification of aetiologies made on the basis of cytological examination of the CSF, specific colony morphology on blood and or chocolate agar, staining properties on Gram stain or by detection of specific antigens in the CSF by latex agglutination test or a rapid diagnostic test,^22^ and the metagenomic protocol for use with molecular methods.^23^ There were 10 articles that fell within this category with prevalence rates ranging from 1.7% to 20.4%.^24,25,26,27,28,29,30,31,32,33,34,35,36,37,38,39,40,41,42,43,44^ Two studies also utilised the molecular approach in the identification of bacterial aetiologies.^25,33^

The second category of studies included those that reported the varying serogroups of *N. meningitidis*, as well as the serotypes of *H. influenzae* and *S. pneumoniae* associated with meningitis in Nigeria. Between 2008 and 2021, 13 studies reported *N. meningitidis* serogroups A, B, C, W135, X, and Y as responsible for CSM infections in northern Nigeria. Seven of these studies^12,25,34,35,36,37,38^ reported *N. meningitidis* serogroup C as the only pathogen, while one study^39^ implicated *N. meningitidis* serogroup A. Two studies^40,41^ implicated both *N. meningitidis* serogroups A and C. One study conducted in 2016 reported *N. meningitidis* serogroups A, C, and W135,^32^ while another study^42^ reported four serogroups (*N. meningitidis* serogroups A, B, C, and W135) as the circulating serogroups in Jigawa State, northern Nigeria. The last study^33^ reported all six serogroups of *N. meningitidis*, including serogroups A (*sacB*), B (*synD*), C (*synE*), W135 (*synG*), X (*xcbB*), and Y (*synF*), as well as the not-typeable (non-groupable or serogroup-negative) strains during the 2017 and 2018 meningitis seasons.

For *H. influenzae,* Nnadi and colleagues^41^ reported *H. influenzae* type b as the only serotype implicated in their study, while another study^33^ reported encountering five serotypes of *H. influenzae*, including serotypes a (*acsB*), b (*bcsB*) c (*ccsD*), e (*ecsH*), and f (*bexD*).

For *S. pneumoniae,* one study^25^ implicated *S. pneumoniae* serotypes 1, 5, and 19F, while another study^43^ reported *S. pneumoniae* serotypes 6, 19, and 20. The last study^33^ reported *S. pneumoniae* serotypes 1 (*Wzy1*), 4 (*Wzy4*), 5 (*Wzy5*), and 9 (*Wzy9*). From these three studies,^25,33,43^ the *S. pneumoniae* serotypes circulating in northern Nigeria are serotypes 1 (*Wzy1*), 4 (*Wzy4*), 5 (*Wzy5*), 6 (*WciP*), 9 (*Wzy9*), 19 (*Wzy19*), 19F (*Wzy19F*), and 20 (*Wzy20*).

In Nigeria, therefore, the circulating *N. meningitidis* serogroups are *N. meningitidis* A (*sacB*), B (*synD*), C (*synE*), W135 (*synG*), X (*xcbB*), and Y (*synF*);^12,25,32,33,34,35,36,37,38,39^ *H. influenzae* serotypes a (*acsB*), b (*bcsB*), c (*ccsD*), e (*ecsH*), and f (*bexD*);^33,38^ and *S. pneumoniae* serotypes 1 (*Wzy1*), 4 (*Wzy4*), 5 (*Wzy5*), 6 (*WciP*), 9 (*Wzy9*), 19 (*Wzy19*), 19F (*Wzy19F*), and 20 (*Wzy20*).^25,33,43^

The third category of articles were those that reported the circulating invasive genotypes of *N. meningitidis, H. influenzae*, and *S. pneumoniae* implicated in meningitis outbreaks in northern Nigeria. There were three studies in this category. *N. meningitidis* genotypes *abcZ, adk, aroE, fumC, pdhC*, and *pgm*;^44^ *H. influenzae* genotypes *adk, fucK*, and *mdh*;^45^ and *S. pneumoniae* genotypes *aroE* and *gki*^46^ have been reported to be circulating among CSM patients in parts of northern Nigeria.

The final category comprises articles that reported vaccination during meningitis outbreaks (reactive vaccination strategy) and those that reported vaccination before any meningitis outbreak (preventive vaccination strategy). Thirteen articles discussed the reactive vaccination strategy, whereas 21 studies discussed the preventive vaccination strategy.

The reactive vaccination strategy has been faulted by some authorities owing to several identified major drawbacks. (1) There are response delays due to challenges with confirming the outbreak, rapidly deploying the vaccines, and organising the vaccination campaigns.^47^ (2) There are also gaps associated with laboratory confirmation during outbreaks.^47^ (3) Limited and late laboratory confirmation is a bottleneck in the submission of International Coordinating Group (ICG) vaccine requests and decision-making on vaccine release.^48^ (4) Another identified drawback of the reactive vaccination strategy is that delays in vaccine deployment and campaign planning hamper response.^47^ (5) In 2012^49^ and 2015,^50^ limited emergency stockpiles undermined the timeliness and effectiveness of outbreak response. (6) There was also the difficulty in accessing some of the more rural and remote communities experiencing the outbreak, which hampered early outbreak response activities.^41^ (7) Healthcare workers’ limited capacity for CSF specimen collection is another drawback.^41^ (8) There is also the certainty of between 8% and 15% deaths during epidemics even when the disease is diagnosed early and adequate treatment is started.^51^ (9) At the end of every meningitis season, 21% – 40% deaths are being recorded.^52,53^ (10) Another major drawback is the unlikely reduction of the number of epidemic cases by more than half with the commencement of mass vaccination after an outbreak.^54,55^ (11) About 10% – 30% of survivors of CSM have sequelae, such as hearing loss, neurological disability, impaired cognitive function, or loss of a limb.^51,53,56,57^

The preventive vaccination strategy is exemplified in the routine Hib childhood vaccination programmes implemented in England and Wales, following which Hib is no longer a major cause of acute bacterial meningitis in children.^58,59,60^ Following the introduction of the different Hib immunisation strategies over the past decades globally, cases in toddlers, older children, and adults have continued to decline rapidly and have now become extremely rare.^61^ Generally, the Hib conjugate vaccine remains highly effective in preventing invasive disease in young children.^62,63^ The experiences gained over the years from the Hib conjugate vaccination programme – the first conjugate vaccine to be introduced in the United Kingdom – and the success of the vaccine in controlling what was once a devastating infection in young children have already contributed to the successful implementation of other conjugate vaccination programmes such as those against invasive meningococcal capsular group C and pneumococcal disease.^64^

Effective vaccines are available for *N. meningitidis* serogroups A, C, W, and Y^65^ and vaccines for serogroup B have been approved for usage since 2015.^66^ In the United States, the quadrivalent ACWY vaccine (meningococcal conjugate effective against serogroups A, C, W, and Y) was recommended for use in persons within the 11–12 years age bracket.^67^ A 2011 update from the Advisory Committee on Immunization Practices (ACIP) recommended administration of a booster dose at age 16.^68^ The Joint Committee on Vaccination and Immunization of Public Health England recommended that individuals aged 14–18 years should be vaccinated with the quadrivalent conjugate ACWY vaccine with the goal of generating herd immunity in the overall population against serogroup W disease.^69^

Following an outbreak of invasive meningococcal disease at a Korean military training centre in 2011, the Korean health authorities recommended that beginning in 2012, new military recruits should receive the quadrivalent ACWY conjugate vaccine before reporting at the training centre.^70^ This is a clear demonstration of using the preventive vaccination strategy against outbreak of meningococcal disease. In the United States, the incidence of invasive meningococcal disease associated with serogroups C, W, and Y among the 11–19-year-old population has been declining since the introduction of the quadrivalent vaccine.^71^

In the United Kingdom, administration of the serogroup C conjugate vaccine was reported to have led to the reduction in serogroup C carriage in university students for up to two years after vaccine administration.^72^ In Burkina Faso, carriage studies two years after widespread use of the serogroup A conjugate vaccine showed that carriage of serogroup A had been nearly eliminated.^73^ The introduction and widespread use of meningococcal vaccines for serogroup C in Europe and for serogroups A, C, W, and Y led to reductions in invasive meningococcal disease associated with these serogroups.^74^

## Discussion

Immunisation programmes against the major CSM bacterial aetiologies (*N. meningitidis, H. influenzae*, and *S. pneumoniae*) have been successfully implemented in Africa, the Americas, Asia, Australia, and Europe.^75,76,77^ Two types of vaccination programmes are being implemented globally: the reactive vaccination strategy or programme and the preventive vaccination strategy or programme. In Nigeria, it is the reactive vaccination strategy that is been practised against *N. meningitidis* outbreak.^78^

The reactive vaccination strategies currently used globally, especially in the African meningitis belt, rely on the detection and timely reporting of suspected meningitis cases beyond a set threshold.^79^ The process involved in identifying and confirming the outbreak, a requirement for requesting vaccine from the ICG, inevitably leads to delays in the overall public health response.^80,81^ Even when the disease is diagnosed early and adequate treatment is started, about 8% – 15% of patients die, often within 24 h – 48 h after the onset of symptoms, or development of sequelae such as brain damage, hearing loss, neurological disability, or impaired cognitive function may occur in 10% – 30% of survivors.^51,53,56,57^ Between 2011 and 2017, during all outbreaks that occurred within the African meningitis belt (Burkina Faso, Cameroon, Ethiopia, Ghana, Niger, Nigeria, and Togo) where reactive vaccination was conducted for the first time with the use of a polysaccharide vaccine that was effective against serogroups A, C, W or A, C, Y, W for 4.7 million people. To ensure maximum potential impact (e.g., herd immunity or protection) and for monitoring purposes, conjugate vaccines were used under specific defined conditions: vaccination of whole districts, distribution of vaccination cards, coverage survey, and strengthened monitoring.^47^

As the term ‘preventive vaccination’ implies, this strategy intends to prevent a meningitis outbreak using vaccination. Preventive medicine and public health share common goals, such as promoting general health, preventing specific diseases, and applying epidemiologic concepts and biostatistical techniques toward these goals. However, preventive medicine seeks to enhance the lives of individuals by helping them improve their own health, whereas public health attempts to promote health in populations through the application of organised community efforts.^82^ The major goal of primary prevention by specific protection involves prevention of specific diseases by using vaccines.^83^

Vaccination against a limited range of serogroups or serotypes of a pathogen can lead to the selection of ‘escape variants’, thus leading to epidemics. For example, the mass vaccination against *N. meningitidis* using a polyvalent polysaccharide vaccine contributed to the selection of non-serogroup C meningococci, leading to outbreaks. Thus, knowledge of genetic diversity is an important prerequisite for the development of successful vaccines.^84^ Epidemiological surveillance is important as it provides the data on which national and regional health authorities can base their vaccination policies. Therefore, to enhance further reductions in the morbidity and mortality associated with invasive CSM, the introduction of serogroup- or serotype-appropriate vaccines should be informed by epidemiologic data.^74^

It should be noted that the commencement of mass vaccination after an outbreak is unlikely to reduce the number of epidemic cases by more than half.^54,55^ This information underscores the need for a paradigm shift from a reactive vaccination strategy to a preventive vaccination strategy on mass vaccination to prevent further CSM epidemics in Africa. Therefore, to ensure successful control of epidemics in the continent, a proper coordination among all the countries within the African meningitis belt is necessary.

It is expected that African countries may incur a high financial burden in rolling out a preventive vaccination programme against meningitis. However, mass vaccination need not be undertaken yearly since several trials have shown that these vaccines may induce adequate antibody responses^57,85^ that may remain high for up to four years in persons aged > 5 years.^86^ Booster vaccination has been recommended for persons who remain at increased risk of meningococcal disease. For individuals whose most recent dose was received at younger than 7 years, a booster dose should be given after three years. However, if the most recent dose was received at age 7 years or older, a booster dose should be administrated after 5 years and every 5 years thereafter as long as the person remains at increased risk for meningococcal disease.^87^ It should be noted that a surveillance and outbreak response system is more effective when the capacity to prevent, detect, and appropriately respond to outbreaks is readily available.^88^

The last explosive CSM epidemic occurred during the 2017 meningitis season. Relying on the documented evidence from Moore in 1992^6^ and the Centers for Disease Control and Prevention in 2011,^7^ and the fact that the yearly meningitis outbreak is punctuated by explosive epidemics occurring every 8–12 years with incidence rates that can exceed 1000 cases per 100 000 population, we forecast the next explosive epidemic in the African meningitis belt to occur between 2024 and 2028.

In order to prevent experiencing the devastations usually associated with CSM outbreaks, and with the identified major drawbacks of the reactive vaccination strategy, we recommend the following: countries within the African meningitis belt should as a matter of responsibility secure appropriate budgetary provisions in their 2023 budget cycle for the procurement of vaccines, medical devices and provision of logistics support to kick-start the preventive vaccination strategy. Donor agencies engaged in the fight against meningitis should key into this preventive vaccination strategy by making funds available for the procurement and the actual administration of relevant and appropriate vaccines before outbreaks. Adequate planning and preparedness against the next explosive CSM epidemic expected to occur, likely from 2024, should commence early in 2023. There should be updated training of medical officers in the process of collection of CSF samples through lumbar puncture from suspected meningitis patients before commencement of antibiotic therapy. The establishment and use of molecular laboratories in the molecular diagnosis (polymerase chain reaction assays) of bacterial and other aetiologies of meningitis is also recommended. There should be regular training and re-training of medical laboratory scientists (biomedical scientists) in the techniques of analysing CSF samples by way of both the phenotypic and molecular methods.

Individual countries within the African meningitis belt should endeavour to utilise the available research results of the circulating serogroups of *N. meningitidis* and serotypes of *H. influenzae* and *S. pneumoniae* within their countries for commencement of the preventive vaccination strategy before the start of the 2024 meningitis season, which normally will commence in December 2023. Effective vaccination is based on the knowledge of the circulating serogroups or serotypes as the case may be. There should be public health education (enlightenment) by relevant authorities to the general local populace on the importance of the preventive vaccination strategy against meningitis. This will enhance general acceptability of the meningitis vaccination programmes. Finally, our recommendations in this review article are relevant for tackling both annual and explosive meningitis outbreaks.
